# Global variations in treatment and outcomes reported for anterior shoulder instability: a systematic review of the literature

**DOI:** 10.1016/j.xrrt.2023.08.005

**Published:** 2023-09-16

**Authors:** Derrick M. Knapik, Andrew W. Kuhn, Aravinda Ganapathy, Joseph T. Gibian, Lauren H. Yaeger, Matthew J. Matava, Matthew V. Smith, Robert H. Brophy

**Affiliations:** a Department of Orthopedic Surgery, Washington University School of Medicine, St. Louis, MO, USA; b Washington University School of Medicine, St. Louis, MO, USA; c Becker Medical Library, Washington University School of Medicine, St. Louis, MO, USA

**Keywords:** Anterior shoulder, Instability, Global variation, Stabilization, Latarjet, Glenoid, Bankart

## Abstract

**Background:**

Anterior shoulder instability is a common problem around the world, with a high risk for recurrence following the index dislocation. Surgical stabilization is commonly indicated for persistent instability, particularly in patients at high risk for recurrence, to minimize the risk of further labral injury and glenoid bone loss. However, there is little known about global geographic differences in the surgical management of anterior shoulder instability. As such, the purpose of this study was to evaluate and systematically review regional differences in the surgical treatment of anterior shoulder instability, particularly the indications for and outcomes from bony stabilization procedures compared to soft tissue procedures.

**Methods:**

A systematic review, in accordance with the 2020 Preferred Reporting Items for Systematic Reviews and Meta-Analyses guidelines, was performed. Inclusion criteria consisted of level I and II evidence studies evaluating indications, techniques, and outcomes following operative management of anterior shoulder instability published from January 2000 to September 2021. Studies meeting inclusion criteria were grouped into four global regions (Asia, Europe, North America, South America) based on primary study location. Patient demographics, procedural details, patient reported outcomes, and complications (recurrence and reoperation rates) were compared between regions.

**Results:**

Sixty (n = 60) studies (5480 patients) were identified. Eighty-six percent of all patients were male, with a mean age of 26.7 years. There was no difference in mean patient age, though patients undergoing bony stabilization procedures were older than those undergoing soft-tissue stabilization procedures (*P* = .0002) in all regions. The proportion of bony versus soft-tissue procedure groups did not differ significantly among regions. The indications for bony stabilization procedures varied significantly. Mean final follow-up was 3.5 years. Recurrent instability was 5.0% and did not vary across regions. However, recurrent instability occurred more frequently following soft-tissue compared to bony stabilization procedures (*P* = .017). South American studies utilized fewer anchors during soft tissue stabilization (*P* < .0001) and reported a higher reoperation rate compared to other regions (*P* = .009).

**Conclusion:**

There is global variation in the reporting of outcomes following surgery for anterior shoulder instability. The proportion of bony and soft-tissue procedures is similar, irrespective of geographic region. Recurrent instability does not vary by region but occurs more frequently following soft-tissue compared to bony stabilization procedures. There are a number of potential medical and nonmedical factors that may affect global variation in the surgical treatment of anterior shoulder instability.

The incidence of anterior shoulder dislocations and recurrent instability has been reported to be 0.08 per 1000 person-years in the United States, with 1.69 dislocations per 1000 person-years in the United States military population.^22^^,^^51^ Among patients undergoing closed reduction following anterior shoulder dislocation, 19% require repeat closed reduction at a median time of 0.9 years from the initial injury.^38^ Increased odds of recurrent instability and the need for surgical intervention increases by 4.1% and 2.8%, respectively, for every year decrease in patient age.^35^ Recurrent instability increases the risk of rotator cuff and axillary nerve injury, as well as progressive soft tissue and bony injury to the anterior glenoid.^2^ Long-term sequelae of recurrent instability includes the development of glenohumeral osteoarthritis, with 22.7% of patients developing clinically significant glenohumeral joint osteoarthritis at a mean of 15 years following dislocation.^31^

While most primary anterior shoulder instability events in the absence of critical glenoid bone loss or off-track lesions are initially managed nonoperatively, recurrent instability has been reported to occur in 38%-80% of shoulders.^1^^,^^9^^,^^27^, ^28^, ^29^^,^^49^^,^^58^^,^^70^ In the setting of recurrent instability, surgical fixation is often recommended. However, there remains substantial heterogeneity in the surgical approaches utilized for the treatment of anterior shoulder instability. Arthroscopic stabilization procedures have become increasingly common, especially in North America, where it has been reported to comprise up to 90% of soft tissue stabilization procedures.^36^ Soft tissue fixation procedures are often recommended in the setting of subcritical glenoid bone loss. However, recent investigations have shown that the amount of bone loss warranting bony stabilization continues to decrease.^16^^,^^24^^,^^65^^,^^66^ In cases in which instability is attributed to both soft tissue and bone loss, bony stabilization procedures are recommended, with options consisting of the Latarjet and modified Latarjet techniques, tibial plafond allograft, Bristow technique, Trillat technique, and use of tricortical iliac crest autograft.^10^^,^^44^^,^^63^^,^^64^^,^^69^

Despite the availability of data evaluating treatment options and outcome scores following anterior shoulder stabilization procedures, the degree of global variation in the treatment of anterior shoulder instability remains largely unknown. Potential factors that may affect geographic variation in treatment approach may reflect differences in training, experience, cultural beliefs, economic factors, technological access and degree of industry support.

The purpose of this systematic review was to evaluate global differences in the indications, patient demographics, surgical techniques, instrumentation, and patient-reported outcome measures (based on Level I and II evidence studies) in the surgical management of anterior shoulder instability. The authors hypothesized that there would be a higher prevalence of bony stabilization procedures reported outside of North America, with no significant regional differences in reported outcomes or the incidence of complications.

## Methods

A systematic review was conducted in accordance with the 2020 Preferred Reporting Items for Systematic Reviews and Meta-Analyses guidelines.^52^ A medical librarian [L.H.Y] searched the literature for records including the concepts of anterior shoulder instability, surgical intervention, randomized controlled trials (RCTs), cohort, cross-sectional, and case-control studies. Search strategies were created using a combination of keywords and controlled vocabulary in Embase.com 1947- , Ovid Medline 1946- , Scopus 1823- , Cochrane Central Register of Controlled Trials, and Clinicaltrials.gov 1997- . All search strategies were completed on September 14, 2021, and were limited to studies published from January 2000 to September 2021. Fully reproducible search strategies for each database can be found in the Supplement. [Supplementary Appendix 1] A separate independent search was conducted on February 9, 2023, to find any additional trials or comparative trials published to February 2023.

Inclusion criteria included: level I or II studies reporting on the treatment of patients with anterior shoulder instability, written in the English language or with an English-language translation available, published between January 2000 and September 2021, with at least one study arm evaluating operative management of anterior shoulder instability. Level of evidence was determined by the criteria set forth by the Oxford Centre for Evidence-Based Medicine.^53^ Exclusion criteria consisted of: studies in which both arms evaluated nonoperative management (ie, no surgery was performed), studies evaluating posterior or global instability, studies with opioid or other pain medication consumption as the sole outcome, studies in which surgical technique (open versus arthroscopic, soft tissue versus bony procedures) performed were not specified or were heterogeneous, systematic reviews, meta-analyses, or ongoing trials.

Following the initial literature query, two authors [A.G., J.T.G] performed preliminary screening of abstracts. Screened abstracts underwent independent full-text review by two authors [A.W.K., J.T.G.]. Any disagreements in study selection were discussed and decided by a third independent author [D.M.K.].

Following screening, studies meeting inclusion/exclusion criteria were grouped into four global regions based on the primary site of investigation: Asia, Europe, North America, and South America. Of note, studies based in Istanbul, Turkey were classified as European-based or Asian-based depending on specific location of the facility in Istanbul, which covers both continents. Australia was considered part of Asia. The following data were recorded for each study: level of evidence, country of investigation, and study design. Within each study, patients were assigned to one of two study groups based on procedure performed (bony versus soft-tissue procedure). For each study group, patient demographics (age, sex), surgical approach (open vs. arthroscopic), surgical position (beach-chair versus lateral decubitus/supine), fixation method (eg, suture anchors) and number of implants for soft tissue procedures, bone graft source (if applicable) and fixation method (screws, suspensory fixation) for bony stabilization procedures were also recorded. Mean final follow-up, and any patient-reported outcome scores were recorded and compared. Complications, including recurrence of instability (subluxation/dislocation), and reoperations were also recorded. Descriptive statistics, parametric and nonparametric tests, and pooled meta-analyses utilizing random effect models were performed utilizing SPSS software (IBM Corp., Armonk, NY, USA) at the 95% confidence interval (CI) level.

## Results

**Figure 1:**
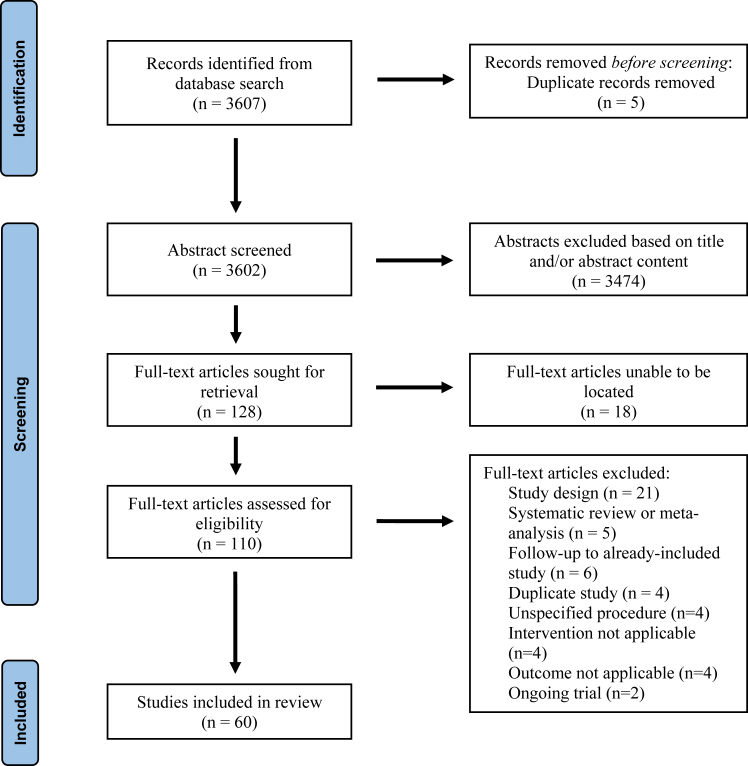
Preferred reporting items for systematic reviews and meta-analyses (PRISMA) flowchart of study.

Following abstract screening, 110 full-text articles were available for review. A total of 60 studies, consisting of 5480 patients, were identified meeting inclusion/exclusion criteria and included for analysis (Fig. 1).

### Study characteristics

**Figure 2:**
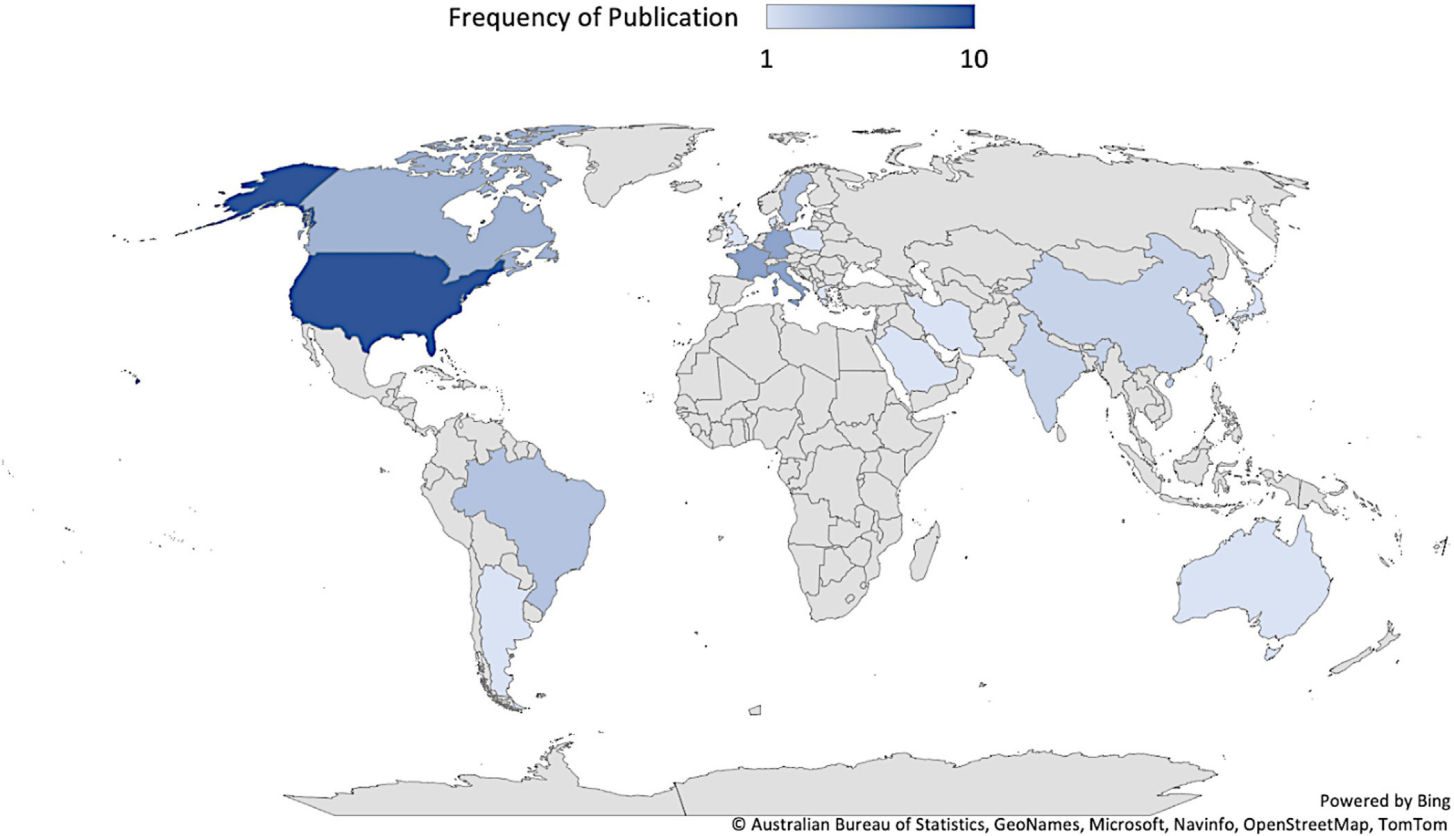
Frequency of publications by country.

The United States (n = 10; 16.7%),^4^^,^^5^^,^^8^^,^^11^^,^^15^^,^^17^^,^^40^^,^^50^^,^^59^^,^^68^ France (n = 5; 8.3%),^41^^,^^46^^,^^48^^,^^55^^,^^67^ Germany (n = 5, 8.3%),^19^^,^^23^^,^^26^^,^^43^^,^^45^ and Italy (n = 5; 8.3%)^13^^,^^21^^,^^42^^,^^54^^,^^60^ were the most represented countries that published studies. Of the 60 included studies, there were 14 (n = 14; 12.8%) bony stabilization procedure groups and 95 (n = 95; 87.2%) soft-tissue procedure study groups. The proportion between bony versus soft-tissue procedure groups did not differ among regions. [χ^2^ (df = 3, N = 109) = 4.80, *P* = .19] Complete country publication data is available in Figure 2 and Supplementary Table S1.

There were 28 (n = 28; 46.7%) level I and 32 (n = 32; 53.3%) level II evidence studies included. There was no difference among regions for levels of evidence published. [χ^2^ (3, N = 60) = 3.47, *P* = .32]. An almost equal number of randomized trials (n = 31; 51.7%) and cohort studies (n = 29; 48.3%) were included. Study design was also not found to vary by region. [χ^2^ (3, N = 60) = 2.55, *P* = .47]

### Patient demographics

The median number of patients/shoulders per procedure study group was 34.0 (interquartile range (IQR) 24.0-45.0). The total number of patients per study group varied significantly by region, [F(3,105) = 2.98, *P* = .04] with North American study groups possessing significantly more patients than European (*P* = .02), Asian (*P* = .02), and South American (*P* = .02) study groups. Overall, the number of patients in soft tissue and bony stabilization procedure study groups was not different. [F (1,108) = 0.389, *P* = .534] (Table I).

**Table I:** Median number of patients per region and procedure study groups.

Region (median, IQR)	Soft tissue	Bony	Total
Europe	33.0 (24.0-45.0)	43.8 (25.0-60.5)	31.5 (23.0-45.8)
North America	38.0 (29.0-98.0)	89.0 (89.0-89.0)	39.0 (28.8-98.0)
Asia	35.6 (24.0-42.0)	44.0 (31.5-45.0)	37.0 (24.0-44.0)
South America	25.0 (22.0-25.0)	20.5 (19.0-22.0)	23.5 (19.8-25.0)
Total	34.0 (25.0-45.0)	30.0 (20.0-52.5)	34.0 (24.0-45.0)

*IQR*, interquartile range.

**Figure 3:**
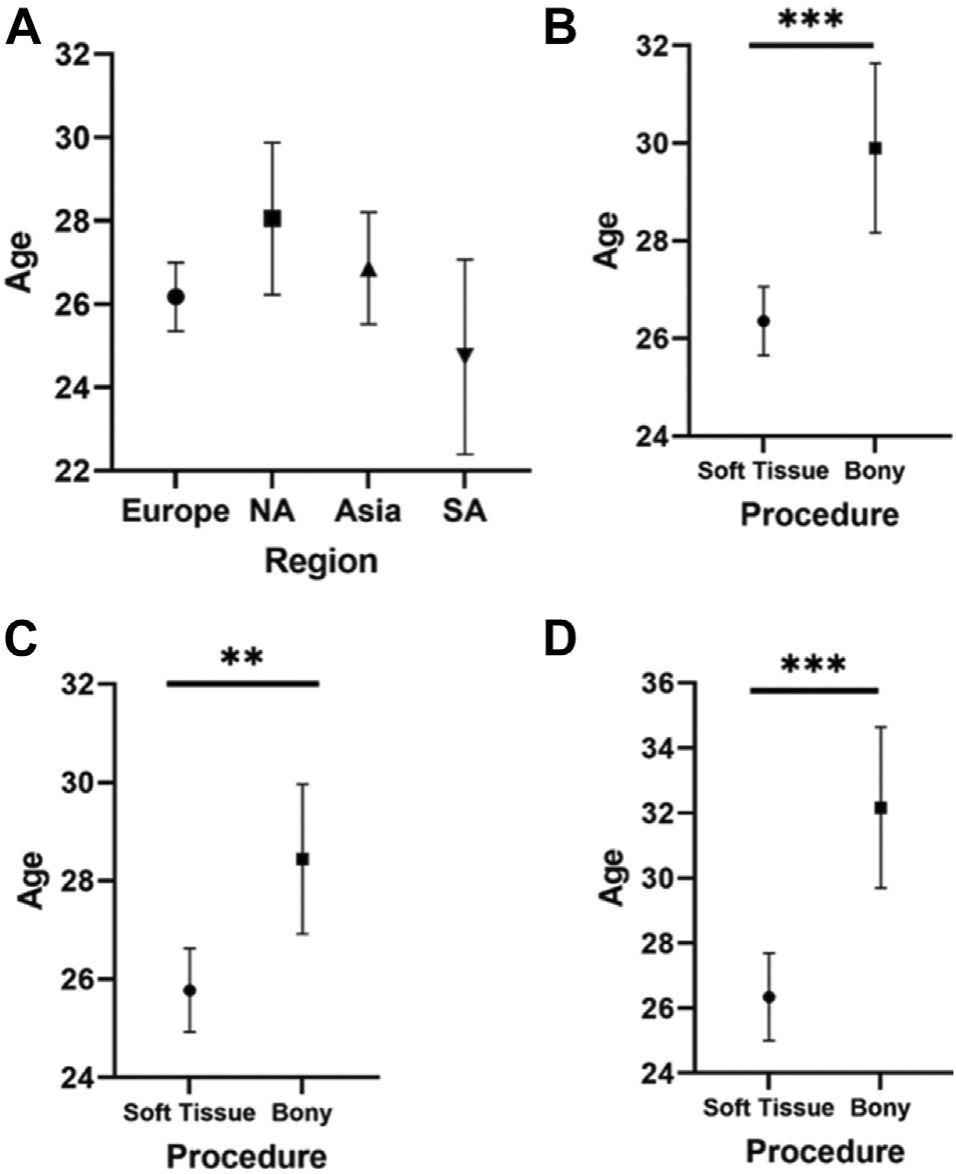
(**A**) Pooled mean ages and 95% confidence intervals among regions, (**B**) procedures, (**C**) between procedures within Europe, and (**D**) between procedures within Asia.

The pooled mean age for all patients was 26.7 (95% CI 26.0-27.4) years, with no difference in mean patient age across regions. (Q_M_ = 5.83, df = 3, *P* = .12) (Table II). There was a significant difference in age across procedures, with patients undergoing bony procedures [29.9 (95% CI 28.2-31.6)] being significantly older than those undergoing soft-tissue procedures [26.4 (95% CI 25.7-27.1]. (Q_M_ = 13.7, df = 1, *P* = .0002). This difference in age was significant in both European (Q_M_ = 9.00, df = 1, *P* = .003) and Asian studies. (Q_M_ = 16.4, df = 1, *P* = .0001) (Fig. 3, *A*-*D*).

**Table II:** Pooled average age of patients by region and procedure study groups.

Region (pooled mean, SD)	Soft tissue	Bony	Total
Europe	25.8 (24.9-26.6)	28.4 (26.9-30.0)	26.2 (25.4-27.0)
North America	28.1 (26.2-29.9)	NS	28.1 (26.2-29.9)
Asia	26.3 (25.0-27.7)	32.2 (29.7-34.6)	26.9 (25.1-28.2)
South America	24.7 (22.4-27.1)	NS	24.7 (22.4-27.1)
Total	26.4 (25.7-27.1)	29.9 (28.2-31.6)	26.7 (26.0-27.4)

*NS*, no studies with available data/patients; *SD*, standard deviation.

The median percentage of females included across all studies was 13.8% (IQR 6.8%-21.0%). The difference in proportion of females included across regions [F (3,88) = 1.039, *P* = .379] and stabilization procedure performed [F (1,90) = 0.007, *P* = .932] was not different (Table III).

**Table III:** Median percentage of females included by region and procedure study groups.

Region (median %, IQR)	Soft tissue	Bony	Total
Europe	16.0% (10.5%-25.0%)	19.4% (6.7%-25.0%)	16.3% (10.3%-24.0%)
North America	17.5% (6.9%-21.3%)	6.9%^a^	16.7% (6.9%-12.3%)
Asia	11.9% (2.7%-19.8%)	21.7% (0.0%-24.5%)	11.9% (2.6%-20.7%)
South America	10.5% (4.4%-18.0%)	NS	10.5% (4.4%-18.0%)
Total	13.3% (7.1%-20.8%)	19.4% (6.7%-24.5%)	13.8% (6.8%-21.0%)

*NS*, no studies with available data/patients; *IQR*, interquartile range.

a Single study with available data.

The median percentage of dominant-side shoulders included was 58.8% (IQR 48.5%-65.1%). Asia was the only country to include data on hand dominance across bony stabilization procedure groups. The overall difference in percentage of dominant-sided shoulders included varied significantly across regions [F (3,48) = 4.87, *P* = .005], with North American studies reporting a significantly higher proportion of nondominant shoulders undergoing stabilization compared to all other regions (*P* < .05). No difference in dominance was appreciated when comparing bony versus soft tissue procedures overall. [F (1,50) = 0.104, *P* = .749] (Table IV).

**Table IV:** Percentage of dominant side included by region and procedure.

Region (median %, IQR)	Soft tissue	Bony	Total
Europe	57.9% (49.6%-67.5%)	NS	57.9% (49.6%-67.5%)
North America	43.8% (33.3%-48.3%)	NS	43.8% (33.3%-48.3%)
Asia	59.5% (50.0%-66.7%)	62.2% (59.1%-63.9%)	59.5% (51.5%-65.9%)
South America	61.5% (57.1%-89.5%)	NS	61.5% (57.1%-89.5%)
Total	58.4% (59.1%-64.7%)	62.2% (59.1%-63.9%)	58.8% (48.5%-65.1%)

*NS*, no studies with available data/patients; *IQR*, interquartile range.

### Procedures performed

**Figure 4:**
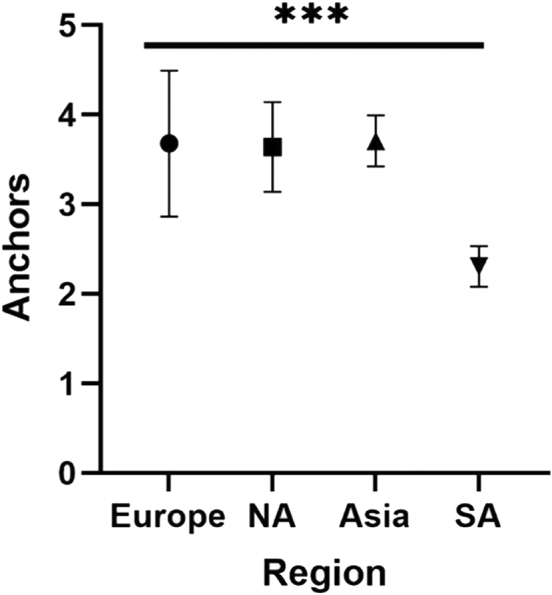
Pooled means of number of anchors utilized for soft tissue stabilization procedures by region.

The most common soft-tissue procedure performed across all regions and soft-tissue stabilization procedure groups was an arthroscopic labral (Bankart) repair (n = 78/95; 82.1%), of which 41% (n = 32/78) involved concomitant procedures such as a capsular shift or Superior Labral Anterior to Posterior (SLAP) lesion repair. When reported, 51% (n = 33/65) of patients were treated in the supine/lateral position (n = 33; 50.8%). There was no difference when comparing beach chair versus supine/lateral positioning for soft-tissue procedures between regions. [χ^2^ (3, N = 65) = 0.205, *P* = .977] The mean number of anchors utilized for soft-tissue stabilization procedures was 3.6 (95% CI 3.2-3.9). South American studies utilized significantly fewer anchors (2.3 [95% CI 2.1-2.5]) when compared to all regions including Europe (3.7 [95% CI 2.9-4.5]), North American (3.6 [95% CI 3.1-4.1]), and Asia (3.4 [3.4-4.0]) (Q_M_ = 68.22, df = 3, *P* < .0001) (Fig. 4).

Of the 14 bony stabilization procedure groups, the most common stabilization procedures performed were open (n = 6; 42.9%)^3^^,^^41^^,^^45^^,^^48^^,^^60^^,^^71^ and arthroscopic Latarjet (n = 3; 21.4%)^41^^,^^48^^,^^71^ coracoid transfer procedures. While 66% (n = 2/3) of arthroscopic Latarjet groups were European,^41^^,^^48^ there were no differences in arthroscopic versus open bony stabilization procedures among regions, overall. [χ^2^ (3, N = 14) = 1.131, *P* = .770] Positioning was available for 5 procedure groups,^3^^,^^41^^,^^60^ with 40% (n = 4/5) of studies reporting use of beach chair positioning and supine positioning reported in 1 study.^41^ The percent glenoid bone loss warranting bony stabilization was reported in 5 studies,^3^^,^^45^^,^^60^^,^^71^ ranging from no bone loss to >20% glenoid loss. In one study from Europe, an instability severity score >3 was used to indicate patients for bony stabilization.^41^ In all studies, the exact amount of glenoid bone loss warranting bony stabilization versus soft-tissue fixation was not explicitly reported. Rather, bone loss in these studies was often treated as an inclusion or exclusion criterion.

### Outcomes

Mean final follow-up was 3.5 years (95% CI 2.8-4.2), which did not vary by region (Q_M_ = 1.67, df = 3, *P* = .64) or procedure (Q_M_ = 0.49, df = 1, *P* = .48) (Table V). The Rowe score was the most common outcome measure reported in all regions except for North America, where the American Shoulder and Elbow Surgeons score (ASES) was most frequently reported (Supplementary Table S2). Across studies, recurrent instability was 5.0% (IQR 0.0%-11.3%) and did not vary across regions. [F (3,108) = 0.558, *P* = .644] Overall, soft-tissue stabilization procedures had a significantly higher rate of recurrence compared to bony procedures. [F (1,108) = 5.89, *P* = .017] Subgroup analyses of each region were not able to be performed due to a small number of bony stabilization procedure groups within each region (Table VI).

**Table V:** Mean follow-up in years by region and stabilization procedure.

Region (pooled mean, 95% CI)	Soft tissue	Bony	Total
Europe	3.0 (2.1-3.8)	8.4 (−4.7 to 21.5)	3.8 (1.8-5.8)
North America	3.9 (2.3-5.6)	NS	3.9 (2.3-5.6)
Asia	3.2 (2.6-3.8)	2.7 (1.8-3.5)	3.2 (2.6-3.7)
South America	3.9 (2.4-5.4)	NS	3.9 (2.4-5.4)
Total	3.3 (2.8-3.7)	5.6 (−0.71 to 1.8)	3.5 (2.8-4.2)

*NS*, no studies with available data/patients; *CI*, confidence interval.

**Table VI:** Recurrence rates among regions and procedures.

Region (median %, IQR)	Soft tissue	Bony	Total
Europe	5.7% (2.6%-10.7%)	3.1% (0.0%-6.3%)	5.0% (2.4%-10.0%)
North America	3.7% (0.0%-18.6%)	NS	3.6% (0.0%-18.5%)
Asia	5.3% (2.4%-11.5%)	0.0% (0.0%-0.0%)	5.1% (2.3%-9.1%)
South America	9.9% (0.0%-12.7%)	0.0% (0.0%-0.0%)	4.0% (0.0%-11.9%)
Total	5.6% (0.5%-12.5%)	0.0% (0.0%-5.1%)	5.0% (0.0%-11.3%)

*NS*, No studies with available data/patients; *IQR*, interquartile range.

The reported reoperation rate was low [0.0% (IQR 0.0%-3.6%)] overall, but differed significantly between regions, [F (3,108) = 4.069, *P* = .009], where studies from South America reported a significantly higher reop eration rate compared to European (*P* = .016), North American (*P* = .001), and Asian (*P* = .003) study groups. There was no difference in reoperation rates across soft-tissue and bony procedure groups, overall. [F (1,108) = 0.675, *P* = .413] (Table VII).

**Table VII:** Reoperation rates among regions and procedures.

Region (median %, IQR)	Soft tissue	Bony	Total
Europe	0.0% (0.0%-5.1%)	0.0% (0.0%-4.6%)	0.0% (0.0%-5.0%)
North America	0.0% (0.0%-0.0%)	NS	0.0% (0.0%-0.0%)
Asia	0.0% (0.0%-3.0%)	0.0% (0.0%-0.0%)	0.0% (0.0%-2.3%)
South America	0.0% (0.0%-11.8%)	10.5% (0.0%-15.8%)	0.0% (0.0%-11.8%)
Total	0.0% (0.0%-3.8%)	0.0% (0.0%-3.8%)	0.0% (0.0%-3.6%)

*NS*, no studies with available data/patients; *IQR*, interquartile range.

## Discussion

There was no significant difference in the reporting of soft-tissue versus bony stabilization procedures, overall, or when comparing each global region. Patients undergoing bony stabilization procedures were significantly older and less likely to experience a recurrence when compared to patients undergoing soft-tissue stabilization. The most common soft-tissue procedure reported was an arthroscopic labral (Bankart) repair, with significantly fewer anchors utilized in studies from South America compared to other global regions. There was no indication whether or not the patients included in the studies from South America had smaller labral tears than the other regions. Bony stabilization procedures were more commonly performed using an open approach, with European studies accounted for the majority of arthroscopic Latarjet procedure groups. Reoperations were reported at a significantly higher rate in studies from South America compared to other regions.

Across all regions, patients undergoing bony stabilization procedures were significantly older than those undergoing soft-tissue stabilization. While younger age is associated with increased odds of recurrent instability requiring surgical intervention,^35^ evidence suggests that older age is associated with increased glenoid bone loss in adolescent patients sustaining traumatic glenohumeral instability.^20^ A large multicenter study performed by the Multicenter Orthopaedic Outcomes Network observed that in 545 patients undergoing primary or revision shoulder stabilization that increasing age was associated with increased odds of patients possessing glenohumeral bone and cartilage lesions at the time of shoulder stabilization.^18^ This is consistent with our finding that patients undergoing bony stabilization are older than patients undergoing soft-tissue stabilization. Interestingly, this may also partly explain the lower rate of recurrence after bony stabilization as age is a well-established risk factor for recurrence.^35^ Much more research is needed to better understand how all of these factors interact to determine the optimal indications for and outcomes from soft tissue versus bony stabilization procedures.

The vast majority of anterior shoulder instability patients across the globe are male, with females comprising only 13.8% of patients across all study groups. The low incidence of females requiring anterior shoulder stabilization is comparable to results reported in a large multicenter epidemiologic study from the Multicenter Orthopaedic Outcomes Network group in which only 18% of 863 patients were female.^30^ In their meta-analysis of 30 studies comprising 9829 patients, Goodrich et al observed a significantly higher rate of recurrent instability in males when compared to females following a Bankart repair (*P* = .0239). Prior investigations have identified male patients, especially those aged 10-16 years, to be at substantially greater risk for both primary and recurrent anterior shoulder instability. It is postulated that this increased risk is a result of the predilection for younger males to participate in contact and collision sports.^37^^,^^57^^,^^61^ Determining further anatomic and patient-related factors, including sport participation unique to each global region that may contribute to the elevated risk of anterior shoulder instability in males relative to females, is warranted to establish risk-reduction protocols to protect younger males athletes against labral and glenohumeral injury.^14^

North American studies included a greater proportion of nondominant shoulders undergoing stabilization when compared to other regions. No correlation has been found between laterality of shoulder dislocation and hand dominance in patients with instability requiring shoulder stabilization.^39^ Similarly, patients’ hand dominance has not been shown to be a substantial factor when deciding treatment for recurrent shoulder instability.^34^ This trend deserves further study, but may be due to other factors that vary across the world such as patient- and/or sport-specific demands.

There was no difference across regions with regards to the frequency of soft-tissue versus bony stabilization procedures, although two of the three studies reporting on the use of the arthroscopic Latarjet reconstruction were from Europe. Traditionally performed using an open technique, arthroscopic bony stabilization procedures have become increasingly popular in European countries.^6^^,^^25^^,^^33^^,^^47^^,^^62^ Lafosse et al reported the arthroscopic approach to offer multiple advantages, including more accurate bone graft placement, faster functional recovery with decreased postoperative stiffness, while minimizing soft tissue violation.^32^ However, arthroscopic bony stabilization is often prohibited due to the complexity of the procedure, increased cost, longer operative times, and increased risk for loss of graft fixation, as well as the significant learning curve associated with the procedure.^12^^,^^56^ As such, our findings likely represent a regional training bias, with European surgeons receiving more training on arthroscopic bony stabilization procedures relative to other global regions.

South American studies reported significantly less anchor usage during soft tissue stabilization compared to other regions, while also reporting higher reoperation rates. When evaluating 91 consecutive patients undergoing arthroscopic stabilization, Boileau et al reported that at a mean follow-up of 36 months, multivariant analysis found that patients undergoing repair with 3 or less anchors were at significantly greater risk for recurrent instability (*P* = .003).^7^ This finding may be related to regional variance in the availability of resources but more research is warranted.

This study is not without limitations. Based on our inclusion of only Level I and II evidence studies, it is possible that given the time and resources necessary to perform high-level outcome studies, many lesser quality studies were excluded, leading to a selection bias in the studies included. As such, this investigation may not accurately capture the true regional trends and/or bias in anterior stabilization surgery between global regions. The ability to access and analysis data to better understand techniques, approaches and outcomes using regional registries would likely allow for a more reliable and accurate overview of regional differences; however, registries are not present in certain regions. Furthermore, the high prevalence of studies from North America likely leads to a further selection bias when analyzing and reporting the aggregated data. Indications for soft-tissue versus bony stabilization were infrequently reported, as no studies reported the mean percentage of anterior glenoid bone loss in patients undergoing bony stabilization. Rather, a minimum, maximum, or range of acceptable glenoid bone loss was often reported, with no rationale provided to justify specific cut-off values. The performance of a remplissage procedure was infrequently reported and therefore not included in our analysis due to the level of heterogeneity. Further statistical analyses comparing variables between regions were limited by the small number of studies meeting our inclusion criteria, especially from South America. As the majority of the studies utilized in our analysis were published from academic centers, data may not be generalizable to surgeons operating in smaller communities or nonacademic settings, as there are currently no comprehensive international repositories of surgical data to definitively determine the procedures and techniques that are actually being performed by all surgeons in each global region.

## Conclusion

There is global variation in the reporting of outcomes following surgery for anterior shoulder instability. The proportion of bony and soft-tissue procedures is similar, irrespective of geographic region. Recurrent instability does not vary by region but occurs more frequently following soft-tissue compared to bony stabilization procedures. There are a number of potential medical and nonmedical factors that may affect global variation in the surgical treatment of anterior shoulder instability.

## Disclaimers:

Funding: No funding was disclosed by the authors.

Conflicts of interest: Knapik: Arthrex, Inc: Research support; DJ Orthopaedics: Paid presenter or speaker; Encore Medical: Other financial or material support; Smith & Nephew: Other financial or material support. Matava: Arthrex, Inc: Paid consultant; Breg: Paid consultant; Schwartz Biomedical: Paid consultant. Smith: American Orthopaedic Society for Sports Medicine: Board or committee member; Arthrex, Inc: Paid presenter or speaker; Research support’ Orthopaedic Journal of Sports Medicine: Editorial or governing board. Brophy: American Journal of Sports Medicine: Editorial or governing board; American Orthopaedic Association: Board or committee member; Journal of the American Academy of Orthopaedic Surgeons: Editorial or governing board. The other authors, their immediate families, and any research foundation with which they are affiliated have not received any financial payments or other benefits from any commercial entity related to the subject of this article.
