# Supplementary material for: Global variations in treatment and outcomes reported for anterior shoulder instability: a systematic review of the literature
Source: JSES Rev Rep Tech. 2023 Sep 16;3(4):469–76. doi: 10.1016/j.xrrt.2023.08.005 (PMC10625007; doi:10.1016/j.xrrt.2023.08.005)
Supplement: Supplementary Appendix 1 [file mmc1.docx]

Supplement

**Full Search Strategies**

**Embase**
Date Searched: 9/14/2021

Applied Database Supplied Limits: 2000-2021

Number of Results: 1,994

Full Search Strategy:

('anterior shoulder instability'/exp OR 'anterior glenohumeral instability'/exp OR 'recurrent shoulder dislocation'/exp OR 'shoulder dislocation'/exp OR ((anterior OR shoulder OR humerus) NEAR/4 (‘shoulder instability*’ OR ‘glenohumeral instability’ OR dislocation OR instability OR subluxation OR disarticulation OR luxation)):ti,ab,kw OR ((‘dislocatio humeri’ OR ‘luxatio humeri’) NEAR/3 (habitualis OR recurrens)):ti,ab,kw) AND ('surgery'/de OR 'arthroscopy'/exp OR 'arthroscopic surgery'/exp OR (surgery OR surgeries OR surgical OR operation OR operative OR arthroscop* OR arthroendoscop* OR ‘Bankart repair’):ti,ab,kw) AND ((‘Level of Evidence 1’ OR ‘level of evidence one’ OR ‘Level of Evidence 2’ OR ‘level of evidence two’ OR ‘Level of evidence 3’ OR ‘level of evidence three’):ti,ab,kw OR ('clinical trial'/de OR 'randomized controlled trial'/de OR 'randomization'/de OR 'single blind procedure'/de OR 'double blind procedure'/de OR 'crossover procedure'/de OR 'placebo'/de OR 'prospective study'/de OR 'randomi?ed controlled' NEXT/1 trial* OR rct OR 'randomly allocated' OR 'allocated randomly' OR 'random allocation' OR allocated NEAR/2 random OR single NEXT/1 blind* OR double NEXT/1 blind* OR (treble OR triple) NEAR/1 blind* OR placebo*) OR 'controlled study'/exp OR 'case control study'/exp OR 'controlled clinical trial'/exp OR 'pretest posttest control group design'/de OR 'static group comparison'/de OR 'cohort analysis'/exp OR 'cross-sectional study'/exp OR 'case control study'/exp OR 'hospital based case control study'/exp OR 'population based case control study'/exp OR ((control OR controlled) NEAR/3 (study OR trial*)):ti,ab,kw OR (cohort NEAR/2 (analysis OR analyses OR study OR studies)):ti,ab,kw OR ((‘cross sectional’ OR ‘cross-sectional’) NEAR/2 (design* OR research OR study OR studies)):ti,ab,kw OR ((‘case-control’ OR ‘case control’) NEAR/2 (studies OR study OR matched)):ti,ab,kw) AND [2000-2021]/py

[Note: RCT filter for Embase is derived from the SIGN filter: The Scottish Intercollegiate Guidelines Network (SIGN) Search Filters Web Page. Available from: <http://www.sign.ac.uk/search-filters.html>]

**Ovid Medline**
Date Searched: 9/14/2021
Applied Database Supplied Limits: 2000-2021
Number of Results: 2,142

Full Search Strategy:

(exp Shoulder Dislocation/ OR (exp Shoulder Joint/ and exp Joint Instability/) OR ((anterior OR shoulder OR humerus) ADJ4 (shoulder instability* OR glenohumeral instability OR dislocation OR instability OR subluxation OR disarticulation OR luxation)).ti,ab,kf. OR ((dislocatio humeri OR luxatio humeri) ADJ3 (habitualis OR recurrens)).ti,ab,kf.) AND (*General Surgery/ OR exp Arthroscopy/ OR (surgery OR surgeries OR surgical OR operation OR operative OR arthroscop* OR arthroendoscop* OR Bankart repair).ti,ab,kf.) AND ((Level of Evidence 1 OR level of evidence one OR Level of Evidence 2 OR level of evidence two OR Level of evidence 3 OR level of evidence three).ti,ab,kf. OR (randomized controlled trial.pt. OR controlled clinical trial.pt. OR randomized.ab. OR placebo.ab. OR drug therapy.fs. OR randomly.ab. OR trial.ab. OR groups.ab.) OR exp Case-Control Studies/ OR exp Cohort Studies OR exp Controlled Before-After Studies/ OR exp Cross-Sectional Studies/ OR ((control OR controlled) ADJ3 (study OR trial*)).ti,ab,kf. OR (cohort ADJ2 (analysis OR analyses OR study OR studies)).ti,ab,kf. OR ((cross sectional OR cross-sectional) ADJ2 (design* OR research OR study OR studies)).ti,ab,kf. OR ((case-control OR case control) ADJ2 (studies OR study OR matched)).ti,ab,kf.)

limit to yr="2000 - 2021"

[Note: RCT filter for Ovid-Medline is derived from: Lefebvre C, Manheimer E, Glanville J. Chapter 6: Searching for studies. In: Higgins J, Green S (editors). Cochrane Handbook for Systematic Reviews of Interventions. Version 5.1.0 (updated March 2011). The Cochrane Collaboration, 2011. Available from [www.cochrane-handbook.org](http://www.cochrane-handbook.org/)]

**Scopus**
Date Searched: 9/14/2021
Applied Database Supplied Limits: 2000-2021
Number of Results: 2,015

Full Search Strategy:

((TITLE-ABS-KEY((anterior OR shoulder OR humerus) W/4 (“shoulder instability*” OR “glenohumeral instability” OR dislocation OR instability OR subluxation OR disarticulation OR luxation))) OR (TITLE-ABS-KEY((“dislocatio humeri” OR “luxatio humeri”) W/3 (habitualis OR recurrens)))) AND ((TITLE-ABS-KEY(surgery OR surgeries OR surgical OR operation OR operative OR arthroscop* OR arthroendoscop* OR “Bankart repair”))) AND ((TITLE-ABS-KEY(“Level of Evidence 1” OR “level of evidence one” OR “Level of Evidence 2” OR “level of evidence two” OR “Level of evidence 3” OR “level of evidence three”)) OR ( ( INDEXTERMS ( "clinical trials" OR "clinical trials as a topic" OR "randomized controlled trial" OR "Randomized Controlled Trials as Topic" OR "controlled clinical trial" OR "Controlled Clinical Trials" OR "random allocation" OR "Double-Blind Method" OR "Single-Blind Method" OR "Cross-Over Studies" OR "Placebos" OR "multicenter study" OR "double blind procedure" OR "single blind procedure" OR "crossover procedure" OR "clinical trial" OR "controlled study" OR "randomization" OR "placebo" ) ) OR ( TITLE-ABS-KEY ( ( "clinical trials" OR "clinical trials as a topic" OR "randomized controlled trial" OR "Randomized Controlled Trials as Topic" OR "controlled clinical trial" OR "Controlled Clinical Trials as Topic" OR "random allocation" OR "randomly allocated" OR "allocated randomly" OR "Double-Blind Method" OR "Single-Blind Method" OR "Cross-Over Studies" OR "Placebos" OR "cross-over trial" OR "single blind" OR "double blind" OR "factorial design" OR "factorial trial" ) ) ) OR ( TITLE-ABS ( clinical AND trial* OR trial* OR rct* OR random* OR blind* ) ) ) OR (TITLE-ABS-KEY((control OR controlled) W/3 (study OR trial*))) OR (TITLE-ABS-KEY(cohort W/2 (analysis OR analyses OR study OR studies))) OR (TITLE-ABS-KEY((“cross sectional” OR “cross-sectional”) W/2 (design* OR research OR study OR studies))) OR (TITLE-ABS-KEY((“case-control” OR “case control”) W/2 (studies OR study OR matched)))) AND ( LIMIT-TO ( PUBYEAR , 2021 ) OR LIMIT-TO ( PUBYEAR , 2020 ) OR LIMIT-TO ( PUBYEAR , 2019 ) OR LIMIT-TO ( PUBYEAR , 2018 ) OR LIMIT-TO ( PUBYEAR , 2017 ) OR LIMIT-TO ( PUBYEAR , 2016 ) OR LIMIT-TO ( PUBYEAR , 2015 ) OR LIMIT-TO ( PUBYEAR , 2014 ) OR LIMIT-TO ( PUBYEAR , 2013 ) OR LIMIT-TO ( PUBYEAR , 2012 ) OR LIMIT-TO ( PUBYEAR , 2011 ) OR LIMIT-TO ( PUBYEAR , 2010 ) OR LIMIT-TO ( PUBYEAR , 2009 ) OR LIMIT-TO ( PUBYEAR , 2008 ) OR LIMIT-TO ( PUBYEAR , 2007 ) OR LIMIT-TO ( PUBYEAR , 2006 ) OR LIMIT-TO ( PUBYEAR , 2005 ) OR LIMIT-TO ( PUBYEAR , 2004 ) OR LIMIT-TO ( PUBYEAR , 2003 ) OR LIMIT-TO ( PUBYEAR , 2002 ) OR LIMIT-TO ( PUBYEAR , 2001 ) OR LIMIT-TO ( PUBYEAR , 2000 ) )

[Note: RCT filter for Scopus from the National University of Singapore medical library systematic review guide: http://libguides.nus.edu.sg/c.php?g=145717&p=2470589]

The Cochrane Library

Date Searched: 9/14/2021
Applied Database Supplied Limits: 2000-2021
Number of Results

- CENTRAL 315

Full Search Strategy:

([mh “Shoulder Dislocation”] OR ([mh “Shoulder Joint”] AND [mh “Joint Instability”]) OR ((anterior OR shoulder OR humerus) NEAR/4 (“shoulder instability*” OR “glenohumeral instability” OR dislocation OR instability OR subluxation OR disarticulation OR luxation)):ti,ab,kw OR ((“dislocatio humeri” OR “luxatio humeri”) NEAR/3 (habitualis OR recurrens)):ti,ab,kw) AND ([mh “General Surgery”] OR [mh “Arthroscopy”] OR (surgery OR surgeries OR surgical OR operation OR operative OR arthroscop* OR arthroendoscop* OR “Bankart repair”):ti,ab,kw)

with Publication Year from 2000 to 2021, in Trials

**ClinicalTrials.gov**
Date Searched: 9/14/2021

Number of Results: 31

Full Search Strategy:

“anterior shoulder instability” AND (arthroscopy OR surgery)
