# Supplementary material for: Global variations in treatment and outcomes reported for anterior shoulder instability: a systematic review of the literature
Source: JSES Rev Rep Tech. 2023 Sep 16;3(4):469–76. doi: 10.1016/j.xrrt.2023.08.005 (PMC10625007; doi:10.1016/j.xrrt.2023.08.005)
Supplement: Supplementary Table S1 [file mmc2.docx]

**Supplemental Table S1.** Publication count by country

| **Country** | **Number of Publications (%)** |
| --- | --- |
| Argentina | 1 (1.7%) |
| Australia | 1 (1.7%) |
| Brazil | 3 (5.0%) |
| Canada | 4 (6.7%) |
| China | 2 (3.3%) |
| Denmark | 1 (1.7%) |
| England | 1 (1.7%) |
| France | 5 (8.3%) |
| Germany | 5 (8.3%) |
| Greece | 1 (1.7%) |
| India | 2 (3.3%) |
| Iran | 1 (1.7%) |
| Israel | 1 (1.7%) |
| Instanbul | 2 (3.3%) |
| Italy | 5 (8.3%) |
| Japan | 1 (1.7%) |
| Korea | 2 (3.3%) |
| Poland | 1 (1.7%) |
| Saudi Arabia | 1 (1.7%) |
| Scotland | 1 (1.7%) |
| Singapore | 1 (1.7%) |
| South Korea | 3 (5.0%) |
| Sweden | 3 (5.0%) |
| Taiwan | 1 (1.7%) |
| United Kingdom | 1 (1.7%) |
| United States | 10 (16.7%) |
