# Supplementary material for: Global variations in treatment and outcomes reported for anterior shoulder instability: a systematic review of the literature
Source: JSES Rev Rep Tech. 2023 Sep 16;3(4):469–76. doi: 10.1016/j.xrrt.2023.08.005 (PMC10625007; doi:10.1016/j.xrrt.2023.08.005)
Supplement: Supplementary Table S2 [file mmc3.docx]

**Supplemental Table S2.** Patient reported outcome measures reported by region

| **Region** | **Preoperative Outcome Scores** | **Postoperative Outcome Scores** |
| --- | --- | --- |
| Asia | Rowe: 9  ASES: 6  Constant-Murley: 6  VAS: 4  UCLA: 3  KSSI, Oxford, Tegner: 1 | Rowe: 12  ASES: 7  Constant-Murley: 7  VAS: 5  UCLA: 4  DASH, KSSI, Oxford, Tegner, WOSI: 1 |
| Europe | Rowe: 8  Constant-Murley: 4  WOSI: 3  DASH: 2  Oxford: 2  Walch-Duplay: 2  ASOSS, SF-12, Shoulder Activity Scale, SST, VAS, VR-12: 1 | Rowe: 15  Constant-Murley: 10  Rowe: 8  WOSI: 8  DASH: 6  Oxford: 3  VAS: 3  SST: 2  Walch-Duplay: 2  ASES, ASOSS, SF-12: 1 |
| North America | ASES: 6  WOSI: 6  SANE: 3  SST: 2  Constant-Murley, VAS, VR-12: 1 | ASES: 8  WOSI: 8  SANE: 4  SST: 3  Rowe: 2  VAS: 2  Constant-Murley, DASH, UCLA, VR-12: 1 |
| South America | ASES, ASORS, ASOSS, Rowe, VAS, WOSI: 1 | Rowe: 3  ASOSS:2  VAS: 2  ASES, ASORS, DASH, UCLA, WOSI: 1 |

ASES: American Shoulder and Elbow Surgeons Score; ASORS: Athletic Shoulder Outcome Rating Scale; ASOSS: Athletic Shoulder Outcome Scoring System; DASH: Disabilities of Arm, Shoulder, and Hand Score; EQ-5D: EuroQol Standardized Measure of Health-Related Quality of Life; KSSI: Korean Shoulder Score for Instability; PASS: Pediatric/Adolescent Shoulder Survey; SANE: Single Assessment Numeric Evaluation; SF-12, SF-36: Short Form health survey, 12 & 36 items; SST: Simple Shoulder Test; UCLA: University of California-Los Angeles Scale; VAS: Visual Analog Scale; WOSI: Western Ontario Shoulder Instability Index; VR-12: Veterans RAND 12-Item Health Survey
